# Identifying malignant mesothelioma by a pathological survey using the São Paulo state hospital cancer registry, Brazil

**DOI:** 10.36416/1806-3756/e20230343

**Published:** 2024-04-08

**Authors:** Fabiola Del Carlo Bernardi, Eduardo Algranti, Marisa Dolhnikoff, Clóvis Antônio Lopes Pinto, Ivanir Martins de Oliveira, Ester Nei Aparecida Martins Coletta, Eduardo Caetano Albino da Silva, Adauto José Ferreira Nunes, Donaldo Botelho Veneziano, Carolina Terra de Moraes Luizaga, Ricardo Luiz Lorenzi, Diego Rodrigues Mendonça e Silva, Thais Mauad

**Affiliations:** 1. Departamento de Ciências Patológicas, Faculdade de Ciências Médicas da Santa Casa de São Paulo, São Paulo (SP) Brasil.; 2. Diretoria de Pesquisa Aplicada, Fundação Jorge Duprat Figueiredo de Segurança e Medicina do Trabalho - FUNDACENTRO - São Paulo (SP) Brasil.; 3. Departamento de Patologia, Universidade de São Paulo - USP - São Paulo (SP) Brasil.; 4. Departamento de Patologia, A.C. Camargo Cancer Center, São Paulo (SP) Brasil.; 5. Instituto Nacional de Câncer José Alencar Gomes da Silva - INCA - Rio de Janeiro (RJ) Brasil.; 6. Instituto de Assistência ao Servidor Público Estadual de São Paulo - IAMSPE - São Paulo (SP), Brasil.; 7. Departamento de Patologia, Fundação Pio XII/Hospital de Câncer de Barretos, Barretos (SP) Brasil.; Hospital de Câncer de Barretos, Barretos, SP, Brasil; 8. Laboratório de Patologia, Hospital Amaral Carvalho, Jaú (SP) Brasil.; 9. Setor de Registro Hospitalar de Câncer, Hospital Amaral Carvalho, Jaú (SP) Brasil.; 10. Fundação Oncocentro de São Paulo, São Paulo (SP) Brasil.; 11. Escritório Avançado no Estado de Santa Catarina, Fundação Jorge Duprat Figueiredo de Segurança e Medicina do Trabalho - FUNDACENTRO - Florianópolis (SC) Brasil.; 12. Setor de Registro Hospitalar de Câncer, A.C. Camargo Cancer Center, São Paulo (SP) Brasil.

**Keywords:** Mesothelioma, malignant/pathology, Mesothelioma, malignant/diagnosis, Immunohistochemistry, Registries

## Abstract

**Objective:**

To review the pathological diagnosis of possible cases and/or hidden cases of malignant mesothelioma (MM) between 2000 and 2012 using the Hospital-Based Cancer Registry database in the state of São Paulo, Brazil.

**Methods:**

Possible cases were retrieved by assessing the database. Inclusion criteria were being older than 30 years of age and having ICD-O-3 topography and morphology codes related to MM. A board of expert pathologists reviewed the pathology reports and requested paraffin blocks in cases that demanded revision. After staining with calretinin, D2-40, WT-1 (as positive MM markers) and Ber-EP4 and MOC31 (as negative MM markers), cases were divided and studied independently by a pair of pathologists to confirm or discard the diagnosis of MM.

**Results:**

Our sample comprised 482 cases from 25 hospitals, and 130 needed further histological revision. We received 73 paraffin blocks with adequate material. After board analysis, there were 9 cases with a definitive diagnosis of MM, improving the diagnostic rate in 12%. Two cases of previously diagnosed MM were discarded by review.

**Conclusions:**

Our results confirm that part of MM underdiagnosis and underreporting in Brazil is due to incomplete or mistaken pathological diagnosis.

## INTRODUCTION

Malignant mesothelioma (MM) is the commonest primary cancer of the pleura,^1^ but it also occurs in other mesothelial tissues such as the peritoneum, pericardium, and tunica vaginalis. It is a rare cancer, strongly linked to occupational and environmental asbestos exposure with a long latency period. MM has an attributable fraction (AF) to asbestos of approximately 90% in men, while it is lower in women.^2^ The lower AF in women possibly represents our failure to identify non-occupational exposures in history taking.^3^ It is the fingerprint of asbestos consumption in a given society. Due to its rarity and strong link with asbestos, specific registries were implemented in industrialized countries to improve surveillance, to investigate, and to analyze disease distribution, diagnosis, and compensation.^4^ Between 1993 and 2015, the Italian MM registry (designated ReNaM) compiled 27,356 MM cases, from which 93% were pleural, 6.5% were peritoneal, and the remaining 0.5% was pericardial or in the tunica vaginalis.^5^

In addition to Italy, other countries have established national registries for MM: Australia, France, Germany, the UK, and New Zealand are examples. Recognized as another sound experience, Australia registered 14,271 MM cases between 2000 and 2020: 11,633 (81.5%) and 2,638 (18.5%) were in men and in women, respectively. Restricting cases to those diagnosed between 2010 and 2020, 93.7% of those were pleural, 5.5% were peritoneal, 0.2% occurred in the tunica vaginalis, 0.1% was in mediastinum, 0.1% was pericardial, and 0.5% occurred elsewhere (overlapped or unknown sites).^6^

In Brazil, by linking five health information systems between 1996 and 2017, 2,405 MM-related deaths (as the underlying or the contributing cause of death) were retrieved, of which 74.7% were pleural or unspecified and 17.3% were peritoneal.^7^ Since the 1960s, approximately 9 million tons of chrysotile and small amounts of anthophyllite were produced and about 7 million tons of asbestos were consumed in Brazil.^8^ The production and consumption of asbestos-containing products were mostly concentrated in the southeastern region of the country, particularly in the state of São Paulo.^9^

The underdiagnosis of MM and the underreporting of the deaths of these patients is global.^10^ In Brazil, the small number of records of asbestos-related diseases in vital statistics, its work-relatedness, and the few studies addressing the subject limit the understanding of the burden of those diseases. A compilation of records between 2008 and 2014 from hospital admissions, hospital cancer registries, and compulsory disease registry added one third of MM-related deaths to those registered in the Brazilian mortality database.^11^ Based on death certificates between 1996 and 2010, 976 MM records were found, with a male-to-female ratio of 1.4:1, while the number of deaths increased from 44 in 1996 to 85 in 2010.^12^ Considering the period between 2000 and 2012, a study reported that MM was the underlying cause of death in 929 cases and that the mortality rate in the state of São Paulo was increasing.^9^ Both studies pointed out potential underdiagnosis and/or underreporting, given the small number of deaths and the massive asbestos consumption during the period. To foster suspicion, investigation, and diagnosis of pleural MM, a Brazilian guideline has recently been published.^13^

The pathological diagnosis of MM remains challenging and may contribute to underreporting. Diffuse mesotheliomas involving the pleura, pericardium, and peritoneum are heterogeneous tumors, including three main histological subtypes: epithelioid (60-80%), sarcomatoid-including desmoplastic-(< 10%), and biphasic subtypes (10-15%).^14^ However, in most cases, these subtypes can mimic other secondary neoplasms, especially adenocarcinomas, mainly when examining limited specimens, such as effusion specimens and small tissue biopsies. To distinguish mesothelioma from other tumors, metastases, or primary malignancies, an immunohistochemistry panel comprising pancytokeratin (multiple keratins, such as AE1/AE3) plus a minimum of two positive and two negative mesothelial markers are recommended.^14^ ^,^ ^15^ Some markers have more specificity whereas others have more sensitivity, but none of the antibodies used for the diagnosis of MM are 100% sensitive or specific. It is recommended that the panel should have sensitivity or specificity greater than 80%, and the interpretation of immunostaining should consider the localization of the marker (membrane, nucleus, cytoplasm) and the proportion of positive cells, of which more than 10% has been suggested for cytoplasmic membranous markers.^16^ In addition, negativity for the mentioned mesothelial antibodies does not exclude the diagnosis of pleural MM since 30% of these cases present a “null” phenotype.^17^ When facing complex cases, pathologists should seek for an expert second opinion and refer to national mesothelioma panels,^16^ which do not exist in Brazil.

Given the pathological diagnostic challenges associated with MM and variations in expertise among pathologists, coupled with differences in access to diagnostic tools, our hypothesis was that MM may be underdiagnosed within hospitals participating in the Hospital-Based Cancer Registry (HBCR) in the state of São Paulo. As of June of 2021, the HBCR network in the São Paulo state comprises 77 hospitals, providing coverage across all regions of the state.^18^ Between the years 2000 and 2022, the database recorded a total of 1,159,914 cancer cases.^18^

The objective of this study was to create a pathology board of specialists and review the diagnosis of possible cases and/or hidden cases of MM retrieved from the HCBR database in the São Paulo state between 2000 and 2012 by picking on selected topographies and presenting inconsistencies in the original pathology reports. This study is part of the Interdisciplinary Project on Occupational Exposure to Asbestos and its Health Effects in Brazil that aims at investigating the burden of asbestos-related diseases by using Brazilian health information systems.

## METHODS

### Case search and selection

We identified the potential cases by assessing the HBCR database. State public hospitals were the majority of the facilities in the period between 2000 and 2012. The inclusion criteria were as follows: subjects older than 30 years of age; topographic codes according to the International Classification of Diseases for Oncology, 3rd edition (ICD-O-3): mediastinum, pleura, pericardium, and peritoneum; and ICD-O-3 morphology codes that could be differential diagnoses for MM (Table 1).^19^ For instance, an undifferentiated carcinoma (morphology) of the pleura (topography) was considered a potential case to be reviewed by the pathology board.

**Table 1:** International Classification of Diseases for Oncology, 3rd edition (ICD-O-3)-based topography and morphology codes to identify possible undiagnosed malignant mesothelioma cases.

ICD-O-3	Topography code
C38.1	Anterior mediastinum
C38.2	Posterior mediastinum
C38.3	Mediastinum, part unspecified
C38.4	Pleura
C38.8	Overlapping lesion of heart, mediastinum and pleura*
C48.0	Retroperitoneum
C48.1	Specified parts of peritoneum
C48.2	Peritoneum, unspecified
C48.8	Overlapping lesion of retroperitoneum and peritoneum*
ICD-O-3	Morphology code
M8000/1	Neoplasm, uncertain whether benign or malignant
M8000/3	Neoplasm, malignant
M8010/2	Carcinoma in situ, NOS
M8010/3	Carcinoma, NOS
M8012/3	Large cell carcinoma, NOS
M8020/3	Carcinoma, undifferentiated, NOS
M8031/3	Giant cell carcinoma
M8033/3	Pseudosarcomatous carcinoma
M8050/3	Papillary carcinoma, NOS
M8070/3	Squamous cell carcinoma, NOS
M8140/3	Adenocarcinoma, NOS
M8211/3	Tubular adenocarcinoma
M8230/3	Solid carcinoma, NOS
M8244/3	Composite carcinoid
M8260/3	Papillary adenocarcinoma, NOS
M8313/3	Clear cell adenocarcinofibroma
M8800/3	Sarcoma, NOS
M8804/3	Epithelioid sarcoma
M8980/3	Carcinosarcoma, NOS
M9050/3	Mesothelioma, malignant or NOS
M9051/3	Fibrous mesothelioma, malignant or NOS
M9052/3	Epithelioid mesothelioma, malignant or NOS
M9053/3	Mesothelioma, biphasic, malignant or NOS

NOS: not otherwise specified.

Of the 75 hospitals that composed the registry network in 2012, 55 reported 864 cases within the selected topographies/morphologies. We arbitrarily selected hospitals that reported at least ten registries with the abovementioned criteria, comprising 716 cases. No attempt was made to contact the remaining 30 hospital units.

In sequence, the pathological reports of the selected cases were requested and were reviewed by the pathology board, composed of eight pathologists with expertise in pulmonary and/or oncological pathology.

Each report was classified according to the necessity of revision or not. Cases were confirmed as mesothelioma or non-mesothelioma if immunohistochemistry test results present in the reports had an adequate panel of markers. In all other cases, we requested the corresponding paraffin blocks from each institution to perform the pathological review. In none of the cases we had access to clinical data.

### Pathological review

New sections were performed from the paraffin blocks, which were routinely immunostained with five immunohistochemical markers: calretinin, D2-40, and WT-1 (as positive MM markers), as well as Ber-EP4 and MOC31 (as negative MM markers). Briefly, 5-μm thick sections were deparaffinized, and a 0.5% peroxidase in methanol solution was applied for five minutes to inhibit endogenous peroxidase activity. Antigen retrieval was performed with citrate solution for 20 min. Sections were incubated overnight with antibodies, and 3,3 diaminobenzidine (Sigma Chemical Co., St Louis, MO, USA) was used as a chromogen. The sections were counterstained with Harris hematoxylin (Merck, Darmstadt, Germany). All primary and secondary antibodies were applied to negative and positive controls.

The cases were then sorted out among pathologists, divided into four groups of paired pathologists who received a set of slides (those stained with H&E, and those stained with each of the five immunohistochemical markers), and previous pathological reports with the results of any available immunohistochemistry panel. Cases were duly labeled for a blind reading without knowledge of the original institution. Each pair reviewed independently the cases to confirm or discard the diagnosis of MM.

The discordant cases were digitized for review by the pathologist board using a panoramic scanner (Pannoramic SCAN; 3DHISTECH Ltd., Budapest, Hungary). Due to the COVID-19 pandemic, in-person board meetings could not be performed as previously planned. Each pathologist received the scanned images prior to the synchronous virtual meetings. During the meetings, images were conjunctively reviewed, and a consensus diagnosis was reached.

All reviewed cases were classified as confirmed for mesothelioma; mesothelioma discarded; inconclusive for mesothelioma; and inadequate for analysis. For confirmation of mesothelioma diagnosis, at least two mesothelioma markers had to be positive, and Ber-EP4/MOC31 had to be negative. Cases were considered inadequate when there was no sufficient tumor for analysis or if it contained extensive artifacts. Cases were considered inconclusive when the histological aspect was suspected for mesothelioma, but only one MM marker was positive, or one MM/one carcinoma marker was positive.

### Ethical approval

This study was approved by the Research Ethics Committee of the *Instituto de Saúde Coletiva*, *Universidade Federal da Bahia*, located in the city of Salvador, Brazil (CAAE no. 36547514.9.0000.5030).

## RESULTS

There were 25 public hospitals that had more than 10 registries of confirmed mesothelioma or needing revision in the study period, to which we requested the corresponding pathological reports. Thirteen hospitals had more than 20 cases, and 12 had between 10 and 19 cases diagnosed with MM during the study period. According to the inclusion criteria, 716 cases were selected. Demographic data, topography, and the number of cases diagnosed as MM in the registry database are shown in Tables 2 and 3.

**Table 2:** Demographic data and topography of the 716 cases with topography related to malignant mesothelioma in 25 public hospitals in the São Paulo State Hospital Cancer Registry.

Variable	Result
Cases, n	716
Sex M/F, n	429/287
Age, years (mean)	60
Topography, n	
Mediastinum	171
Peritoneum	118
Pleura	256
Retroperitoneum	171

**Table 3:** Demographic data and topography of the 179 cases with the diagnosis of MM. in 25 public hospitals of the São Paulo State Hospital Cancer Registry.

Variable	Result
Cases, n	179
Sex M/F, n	118/61
Age, years (mean) Male Female	60 60 59
Topography, n	
Mediastinum	1
Peritoneum	33
Pleura	131
Retroperitoneum	13

**Figure 1:**
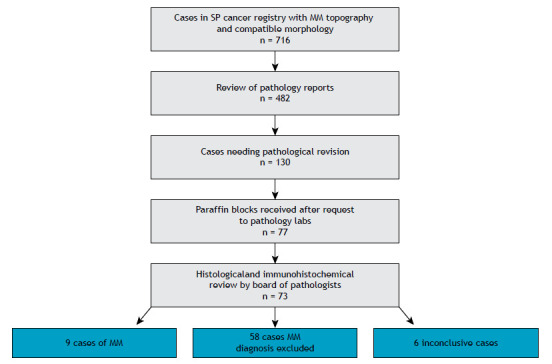
Flow chart showing the different steps of the study. N represents reports and tissue blocks received after request to the corresponding hospitals and pathology laboratories. SP: São Paulo; and MM: malignant mesothelioma.

Figure 1 depicts the study flow chart for the selected 716 cases. After contacting each institution, we received 482 pathology reports from 11 hospitals, all of which from services reporting more than 20 cases and mostly from state referral oncology or university hospitals. The remaining hospitals did not reply, refused to send materials, or had already discarded the blocks. Of the 482 reports, 130 had the diagnosis of MM, and 222 had the diagnosis of MM discarded, since all of the diagnostic criteria were complete in the pathological reports. After analyzing the pathological reports, 130 cases were selected for histological review. After requesting the blocks to the hospitals, we received 77 paraffin blocks, from which 73 had adequate material for analysis. The four discarded blocks were not representative of the tumor or were insufficient to carry out further analyses.

After analysis by the board of pathologists, we had as final diagnoses nine cases of confirmed MM, 58 MM diagnosis discarded, and six cases remained inconclusive. Of the nine confirmed mesothelioma cases, there were three *de novo* diagnoses, and six had the MM diagnosis confirmed by the immunohistochemistry panel that had not been performed before. All MM cases were of epithelioid morphology. Two cases of previously diagnosed mesothelioma were discarded by review. Of the inconclusive cases one had a sarcomatous aspect, three showed epithelioid morphology, and two had an anaplastic aspect. Demographic and topographic of the newly diagnosed or confirmed cases of mesothelioma are shown in Tables 4 and 5.

**Table 4:** Final diagnosis of 73 potential cases with topography and morphology for malignant mesothelioma (MM) with available paraffin blocks for revision.

Variable	Result
Cases, n	73
Sex M/F, n	52/21
Age, years (mean) Male Female	61 59,5 64
Topography, n	
Mediastinum	15
Peritoneum	9
Pleura	28
Retroperitoneum	21
Final diagnosis	
MM	9
Prior diagnosis of MM discarded	2
Inconclusive for MM	6
Discarded for MM	56

**Table 5:** Confirmed cases of malignant mesothelioma after final review of the pathology board.

Case	Age	Sex	Topography	Initial diagnosis
1	55	male	Pleura	Epithelioid Mesothelioma
2	61	male	Retroperitoneum	Sarcoma
3	68	male	Pleura	Epithelioid Mesothelioma
4	59	male	Mediastinum	undifferentiated malignant neoplasm, epithelioid pattern favoring mesenchymal lineage
5	66	male	Pleura	Adenocarcinoma
6	70	female	Pleura	Pleomorphic Carcinoma x mesothelioma
7	68	female	Pleura	Metastatic Adenocarcinoma
8	68	male	Pleura	Undifferentiated malignant neoplasm, suggestive of mesothelioma
9	60	male	Pleura	Epithelioid Mesothelioma

## DISCUSSION

In this study, by reviewing the pathology of cases registered at the São Paulo State HBCR between 2000 and 2012 that presented a topography of MM but inadequate pathological workout, we were able to increase the MM diagnostic rate in 12%, which is substantial considering the rarity of this malignancy. Our results confirm that part of MM underrecognition and underreporting in Brazil is due to incomplete or mistaken pathological diagnosis. To our knowledge, this is the first study on MM in Brazil with a board of specialized pathologists to review cases that could improve MM diagnosis in the country.

Despite our best efforts in contacting hospitals and pathology services, we were unable to retrieve pathological reports or tissue specimens for revision from 14 of 25 pathology laboratories, 12 of which from smaller hospitals that had at least ten cases during the study period. It is not uncommon that smaller and non-academic hospitals make use of third-party laboratories that may change overtime, have no expert pathologists, or have no complete access to immunohistochemistry panels, increasing the chance of misdiagnosing MM cases. Indeed, most of the cases that we investigated came from larger university hospitals or oncological centers with expert pathologists and adequate panels, which make our findings likely to be underrepresented and underestimated. In addition, São Paulo state laws authorize to discard slides and paraffin blocks after five years.^20^ Several steps to overcome this situation should be taken, starting with the creation of a pathology network and financial support for the purchase of antibody panels with the objective of offering expert advice for difficult or suspicious cases. An MM registry, either regional or national, may be a future goal to be pursued, but it demands a complex structure, including integration of the country’s health care systems, a dedicated staff, expert consultancy of hygienists, social service workers, and health professionals, as well as specific and long-lasting financing.^21^

Our data confirm the challenges for the pathological diagnosis of MM and the necessity of clinical information for definitive diagnosis. Even with a board of expert pathologists and recommended immunohistochemistry panels, six cases (8%) remained inconclusive based on pathology alone, as were the cases with sarcomatous or anaplastic aspects, which had inconclusive immunohistochemistry test results.^14^ ^,^ ^15^ A multidisciplinary team is indeed necessary to reach a final diagnosis in difficult cases, especially considering the occupational connection of MM and the legal consequences of an MM diagnosis. In Quebec, Canada, less than a quarter of MM cases identified in the Quebec Tumour Registry were compensated as an occupational disease.^22^ These prompted investigators to study if there was an overregistration of MM cases. An expert panel composed of one pathologist, one radiologist, and one pulmonologist reviewed available materials from cases diagnosed between 2001 and 2002 in provincial hospitals using guidelines defined for pathological diagnosis and/or clinical and radiological data when pathological specimens were unavailable. After analyzing charts with good quality data, they found that 88% of the cases were correctly diagnosed, and MM was not confirmed in 9-11% of the cases. The authors concluded that the provincial registry was a valid source of information.^22^

In this study, two cases previously regarded as MM were discarded after review by the pathology board. One case of pleural MM was considered a metastasis of an adenocarcinoma, and one that had epithelioid features had no positive markers for mesothelioma or adenocarcinoma. These data reinforce that, in the absence of adequate MM panels, the diagnosis must be done with extreme caution.

A major limitation of this study includes its retrospective design. We had no access to clinical and/or radiological information, which could have contributed to diagnostic conclusions. Of the 11 hospitals that sent pathology reports, only two were non-specialized cancer hospitals or university-related services. Also, the lack of access to pathology specimens from 14 hospitals, whose majority (n = 12) were general non-specialized hospitals, may have contributed to an underestimation of MM hidden cases.

In Brazil, asbestos consumption persisted until 2018, and chrysotile mining and exports are still active. Based on consumption data until 2012, it was expected that MM would reach its peak incidence in Brazil by 2026.^8^ Between 1996 and 2017, MM-related mortality was on the rise, faster for men, presenting with a 6% annual mean increase, while it was less than 1% among women.^6^ Part of MM underdiagnosis has its origin during pathological diagnosis, and, therefore, pathologists must be alert to identify MM cases, seeking expert advice and working in a multidisciplinary manner, thereby increasing the quality of cancer registries in this country.
